# Pathogenesis of depression and the potential for traditional Chinese medicine treatment

**DOI:** 10.3389/fphar.2024.1407869

**Published:** 2024-06-25

**Authors:** Weixing Ding, Lulu Wang, Lei Li, Hongyan Li, Jianfa Wu, Jing Zhang, Jing Wang

**Affiliations:** ^1^ College of Traditional Chinese Medicinal Material, Jilin Agricultural University, Changchun, China; ^2^ School of Medicine, Changchun Sci-Tech University, Changchun, China; ^3^ Jilin Provincial International Joint Research Center for the Development and Utilization of Authentic Medicinal Materials, Changchun, China; ^4^ Jilin Province Faw General Hospital, Changchun, China

**Keywords:** depression, pathogenesis hypothesis, antidepressant potential of traditional Chinese medicine, herbal monomers, classical Chinese medicine prescriptions

## Abstract

Depression is a prevalent mental disorder that significantly diminishes quality of life and longevity, ranking as one of the primary causes of disability globally. Contemporary research has explored the potential pathogenesis of depression from various angles, encompassing genetics, neurotransmitter systems, neurotrophic factors, the hypothalamic-pituitary-adrenal axis, inflammation, and intestinal flora, among other contributing factors. In addition, conventional chemical medications are plagued by delayed onset of action, persistent adverse effects, and restricted therapeutic efficacy. In light of these limitations, the therapeutic approach of traditional Chinese medicine (TCM) has gained increasing recognition for its superior effectiveness. Numerous pharmacological and clinical studies have substantiated TCM’s capacity to mitigate depressive symptoms through diverse mechanisms. This article attempts to summarize the mechanisms involved in the pathogenesis of depression and to describe the characteristics of herbal medicines (including compounded formulas and active ingredients) for the treatment of depression. It further evaluates their effectiveness by correlating with the multifaceted pathogenesis of depression, thereby furnishing a reference for future research endeavors.

## 1 Introduction

Major Depressive Disorder (MDD), or depression for short, is a chronic, recurrent, and potentially life-threatening mental disorder that not only reduces people’s quality of life, but also imposes a heavy burden on individuals and their families in terms of health, finances, work, interpersonal relationships, *etc.*, (Sim et al., 2015). MDD is characterized by symptoms such as low mood, cognitive impairment, anhedonia, worthlessness, social phobia, and suicide. Based on the most recent statistics provided by the World Health Organization, it is estimated that over 350 million individuals globally are afflicted by MDD, which accounts for approximately 5% of the global population, making it the fourth most common disease in the world (World Health Organization, 2022). Furthermore, under the influence of the new coronavirus pandemic, the prevalence of depression has risen by 28% (COVID-19 Mental Disorders Collaborators, 2021). In addition, the occurrence of MDD also significantly increases the risk of patients developing other diseases such as cardiovascular disease, stroke, Alzheimer’s disease, and epilepsy (Mathers and Loncar, 2006).

MDD is mainly treated with traditional antidepressants of the monoamine class (López-Muñoz and Alamo, 2009). Such drugs suffer from long onset of action, persistent side effects, and limited therapeutic efficacy (only 50% of patients achieve complete remission) (Deussing and Arzt, 2018) Therefore, it is particularly urgent to find antidepressant drugs that are effective and have fewer side effects. TCM is a natural product with a long history of medication, and it is widely accepted that it is safe and has few side effects. Screening antidepressant formulas and active ingredients from TCM is a promising alternative and may become a major trend in the treatment of MDD in the future. To facilitate research on MDD and the development of new antidepressants, we have compiled an overview of the pathogenesis of MDD and the corresponding therapeutic modalities offered by TCM. This compilation aims to provide a valuable reference for further studies in this field.

## 2 Hypotheses on the pathogenesis of MDD

**FIGURE 1:**
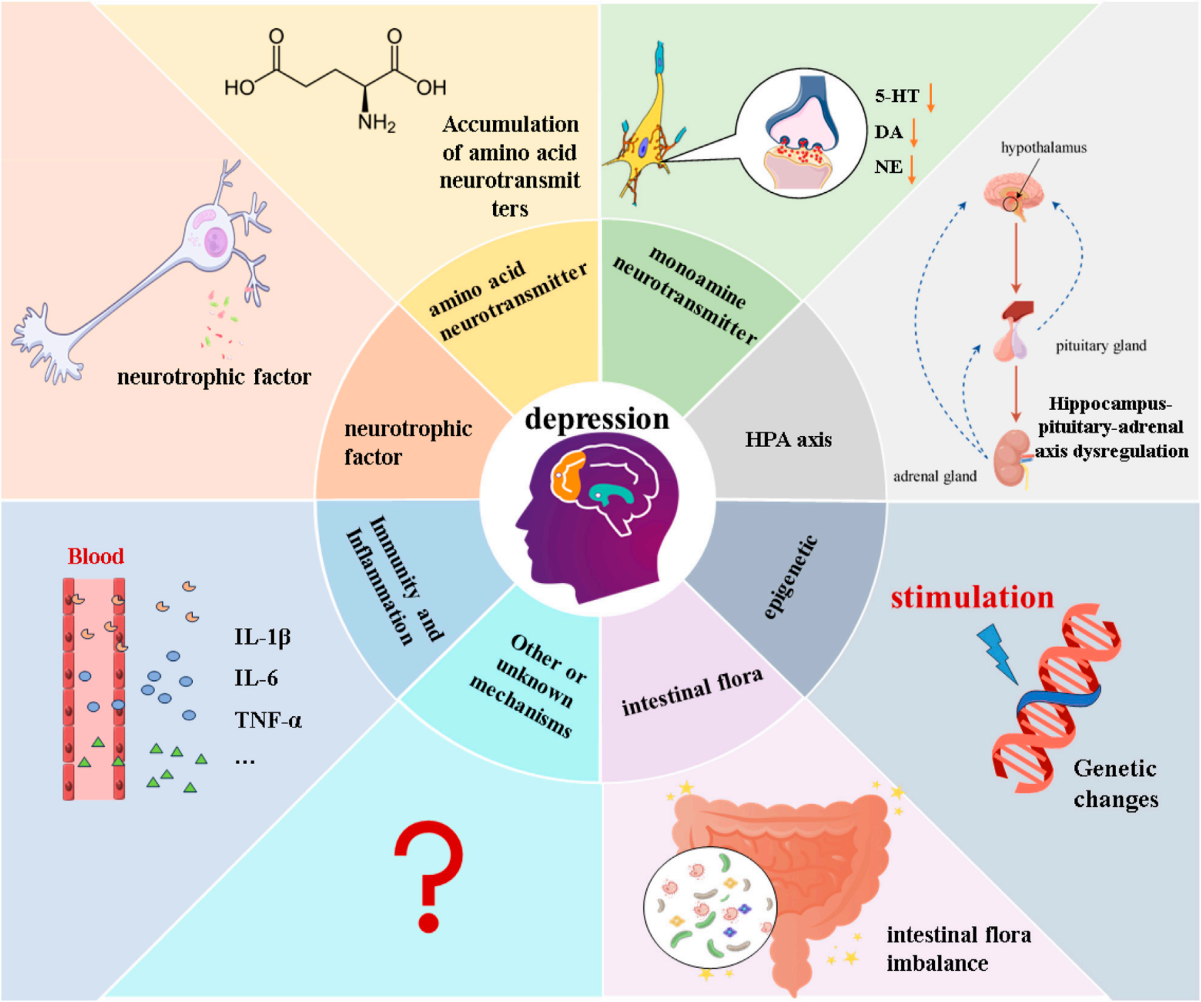
Different hypotheses of the pathogenesis of MDD.

The etiology and pathogenesis of MDD are extremely complex, involving genetic, environmental, biochemical, and psychological factors (Jesulola et al., 2018). Despite numerous in-depth studies, there is no clear concept to explain the causes and mechanisms of MDD development. Based on this, hypotheses explaining the pathogenesis of MDD from various aspects have been proposed, such as epigenetic, monoamine neurotransmitters, Hypothalamus-Pituitary-Adrenal (HPA) axis, neuroplasticity and neurotrophic factors, intestinal microbiota, and inflammatory hypotheses (Dobrek and Głowacka, 2023). Figure 1.

## 3 Antidepressant theory and application of TCM

Chinese medical theory has a long history of understanding MDD-related diseases, and there are many MDD-related diseases recorded in classical Chinese medical texts, such as baihe disease, globus hystericus, renalmass, and depressive psychosis, which belong to the category of “yv zheng” in Chinese medical theory (Zhou, 2007; Yu et al., 2009). According to Chinese medicine theory, MDD is mostly characterized by emotional disorders and stagnation of qi, which leads to a loss of regulation of the liver, a loss of function of the spleen, and a loss of nourishment of the heart. There are internal and external causes for MDD, externally because of emotional factors such as worry, fear, and anger, and internally because the qi of the organs is easily disturbed. The disease is mainly located in the liver, involving the heart, spleen, and kidney; the disease mechanism is mainly stagnation of qi and dysfunction of internal organs. At the beginning of the disease, most of the evidence is solid, due to the stagnation of qi, resulting in food stagnation, phlegm coagulation, blood stasis; as the disease progresses from solid to virtual, it ultimately leads to deficiency of the five viscera, yin and yang imbalance. Common symptoms include liver stagnation, qi stagnation and fire, loss of heart and spirit, deficiency of heart and spleen, and phlegm and qi stagnation (Li and Liu, 2017).

**FIGURE 2:**
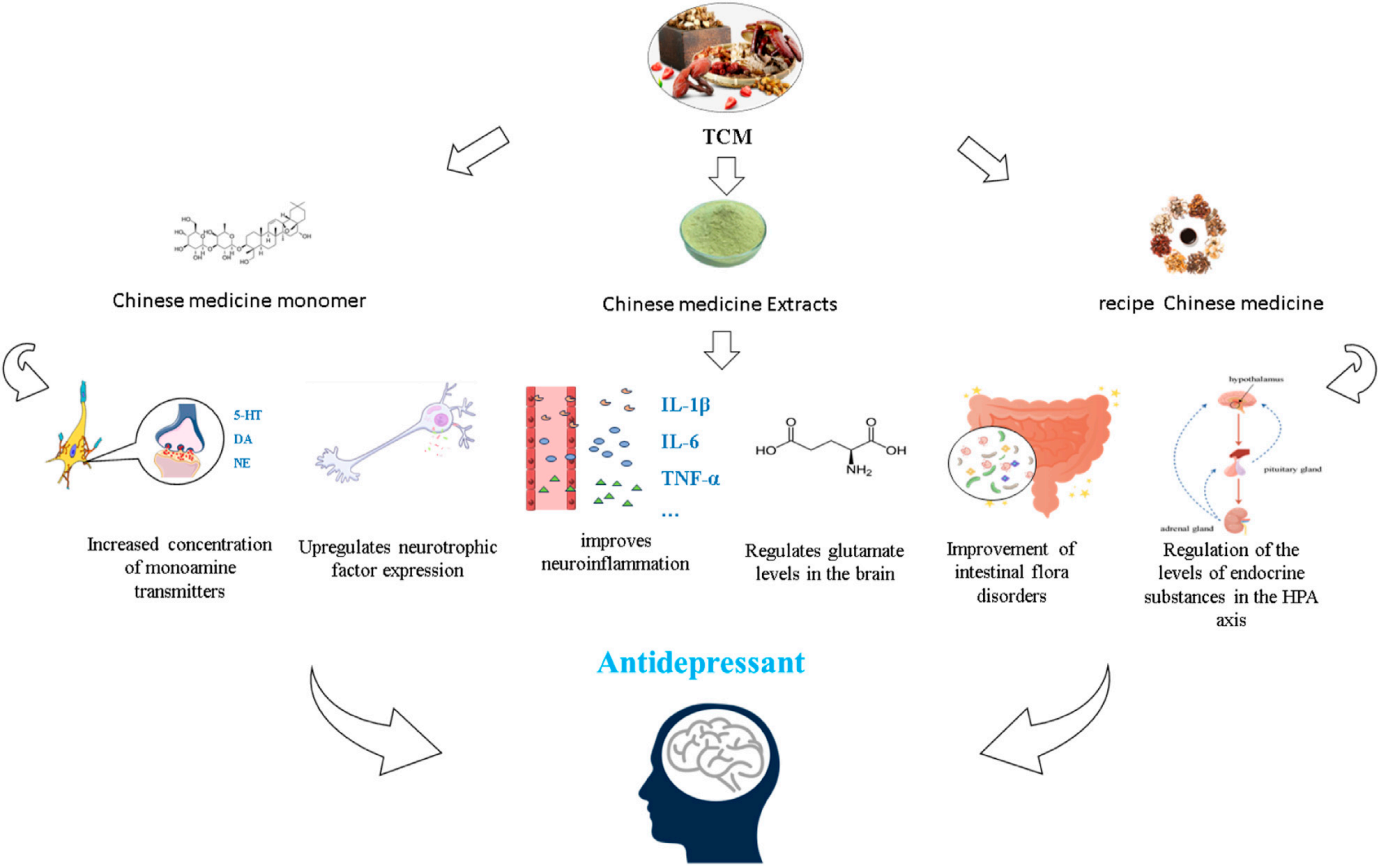
Different forms of TCM exert antidepressant effects based on the hypothesis of the pathogenesis of depression.

With the continuous development of theoretical research on TCM and the advantages of low side effects and low recurrence rate of Chinese medicine, significant progress has been made in the research on TCM for MDD (Yeung et al., 2014). Currently, TCM antidepressant studies are available in the form of monomers isolated from TCM, individual Chinese medicine extracts, and formulas. In this review, we review and discuss the effects of TCM on depression through different mechanisms of action (Figure 2).

## 4 Substance basis of antidepressant efficacy and its mechanism of action in Chinese medicine

With the in-depth exploration of Chinese medicine, many active ingredients or parts with significant antidepressant effects have been found, which can be used as supplements to traditional antidepressants. They can affect depression by modulating monoamine transmitters, glutamate levels, the HPA axis, neurotrophic factors, the immune system, and gut flora.

### 4.1 By modulating monoamine transmitters

The monoamine hypothesis is the first theory in the history of MDD, and this hypothesis suggests that the possible pathogenesis of MDD is a decrease in monoamine neurotransmitter metabolism in the synaptic gap (Schildkraut et al., 1972). Monoamine neurotransmitters are an important class of excitatory neurotransmitters that include two major groups, catecholamines and indoleamines. Catecholamines include norepinephrine (NE) and dopamine (DA), and indoleamines are mainly serotonin (5-HT) (Jovanovic et al., 2022). Many functions in the body are regulated by the monoaminergic system. For instance, anxiety and fear disorders involve 5-HT, which is also related to pain sensitivity control and neurohormonal regulation (Bazian and Grigor’ian, 2006). NE and DA are associated with mood and eroticism, respectively, and are involved in the regulation of selective attentional processes, learning, locomotion, and reward activities (Goddard et al., 2010; Shen et al., 2012). Whereas the monoamine hypothesis is a classic prediction of depression, the underlying pathophysiology of depression is based on decreased levels of monoamine neurotransmitters in the synaptic gap (Delgado, 2000). It has also been found that drugs that act through various mechanisms to increase synaptic monoamine concentrations can improve symptoms of depression (Hirschfeld, 2000). However, it is important to note that the monoamine hypothesis is not without its criticisms. Some researchers argue that the hypothesis oversimplifies the complex etiology of depression and fails to account for other neurotransmitter systems, such as glutamate and GABA, which may also play a role in the development of MDD (Cryan et al., 2008). Additionally, not all individuals with depression respond positively to traditional monoamine-based antidepressant medications, suggesting that other factors may be at play in the development of depression (Pariante, 2017).

Overall, while the monoamine hypothesis has been influential in shaping our understanding of depression and guiding treatment approaches, it is important to continue to research and explore other potential mechanisms involved in the pathology of MDD in order to develop more effective treatments for this debilitating condition. Modulation of synaptic gap monoamine neurotransmitter concentrations and inhibition of monoamine oxidase may be one of the antidepressant mechanisms in Traditional Chinese Medicine (TCM) (Xin-xi, 2011). Research has shown that the active ingredients in TCM can improve the levels of different monoamine transmitters. For example, in a study involving a chronic mild stress (CMS) depressed mouse model, administration of ginkgolide at varying doses (3 mg/kg/d, 6 mg/kg/d, 12 mg/kg/d) was able to increase the levels of dopamine (DA) and serotonin (5-HT) in the hippocampal tissues of the mice and downregulate the levels of monoamine oxidase-A, thereby improving the depression-like behavior of the mice (Yang et al., 2020). Another active ingredient, crocin, found in the Chinese medicine saffron, was also studied in a chronic unpredictable stress (CUMS) mouse model, where crocin at a dosage of 25 mg/kg/d was found to alleviate depressive symptoms by modulating the metabolic level of tryptophan, the precursor of 5-hydroxytryptophan, as demonstrated in metabolomics studies (Luo et al., 2023). TCM active ingredients are known to regulate the levels of different monoamine transmitters through various channels, targets, and levels. For instance, Yuanzhi-1 was found to block synaptic reuptake of monoamine transmitters and simultaneously increase the levels of 5-HT, DA, and norepinephrine (NE) in the synaptic gap.

Monoamine oxidase inhibitors (MAOIs) were one of the first drugs used to treat depression but were phased out because of large side effects (Finberg, 2014). In contrast, a recent study found that curcumin at 50 mg/kg/d could improve the despairing behavior of rifampicin- and buprenorphine-induced mice by acting similarly to MAOIs, whereas curcumin did not have a direct activating effect on monoamines and did not have a significant inhibitory effect on the reuptake of monoamine neurotransmitters (Chen et al., 2006). More studies of herbal active ingredients or extracts to increase the inter-synaptic concentration of monoamines in response to depression are shown in Table 1.

**TABLE 1:** Antidepressant mechanisms of Chinese herbal active ingredients modulating monoamine transmitter levels.

Chemical compound	Source Chinese medicine	Source plant	Traditional efficacy	Pharmacological model	Machine	Reference
Crocin	Stigma croci	*Crocus sativus* L	Activate blood and resolve stasis, dispersing depression and opening up knots	CUMS rat	Improvement of 5-HT precursor tryptophan and lipid metabolism abnormalities	Luo et al. (2023)
Ginkgolide	Ginkgo	*Ginkgo biloba* L	Activate blood and resolve stasis, dredging collaterals and relieving pain, astringing lung QI for relieving asthma	CMS mice Depressed mice after myocardial infarction	Regulates 5-HT, DA levels	Yang et al. (2020)
Saikosaponin A	Radix bupleuri	*Bupleurum chinense DC. or Bupleurum scorzonerifolium Willd*	Harmonize and release the exterior and interior, soothe the liver and resolve constraint, invigorating YING QI	CUMS rat	Increased DA content in the hippocampus	Guo et al. (2020)
Huperzine A	All-grass of Snakefoot clubmoss	*Huperzia serrata* (Thunb. ex Murray) Trevis	Clear heat and resolve toxins, promote tissue regeneration and close wound and stanch bleeding, dissipate (blood) stasis	Post-stroke depression (PSD) rat	Upregulates NE, DA and 5-HT levels in the prefrontal cortex	Du et al. (2017)
Puerarin	Radix puerariae	*Pueraria lobata* (Willd.)Ohwi	Expelling pathogenic factors from muscles for reducing heat, promoting fluid production to quench thirst, promoting eruption, dredging channels and activating collaterals	CUMS rat	Increased levels of 5-HT and 5-HIAA, downregulated levels of CRH, CORT, ACTH.	Qiu et al. (2017)
Yuanzhi-1	Radix Polygalae	*Polygala tenuifolia* Willd. or *Polygala sibirica* L	Calm the nerves and increase intelligence, coordinate the heart and kidney, dispel phlegm, disperse swelling	CMS rat	Increased 5-HT, NE and DA concentrations in the cerebral cortex	Jin et al. (2015)
ginsenoside Rg3	Radix Ginseng	*Panax ginseng* C.A.Mey	Greatly tonify the original qi, restore the pulse and stabilize the body, tonify the spleen and benefit the lungs, engender fluid and nourish blood, calm the nerves and increase intelligence	CUMS rat	Increased 5-HT levels in prefrontal cortex and hippocampus	Xu et al. (2018)
Semen Arecae total phenol	Semen Arecae	*Areca catechu* L	Kill worms, disperse accumulation, move qi, promote urination, interrupt malaria	Forced swimming (FST) and trailing suspended tail (TST) mice	Increased levels of 5-HT and NE.	He et al. (2013)
Curcumin	Rhizoma Curcumae Longae	*Curcuma Longa* L	Removing blood stasis and move qi, inducing menstruation to relieve menalgia	Rifampicin and buprenazine induced despair in mice	Inhibition of monoamine oxidase	Chen et al. (2006)
Resveratrol	giant knotweed rhizome	*Reynoutria japonica* Houtt	Dispelling wind, inducing diuresis, breaking up blood stasis and promoting menstruation	CUMS rat	Increased 5-HT levels and downregulated mRNA expression of 5-HT transporter protein (SERT) in the hippocampus	Shen et al. (2020)
Icariin	Herb of Shorthorned Epimedium	*Epimedium brevicornu* Maxim	Tonifying kidney-yang, strengthening muscles and bones, dispelling wind-dampness	Perimenopausal depression in rats	The expression levels of 5-HT, DA, and NA in brain homogenates were increased by activating the PI3K-AKT pathway	Cao et al. (2019b)
Gentiopicroside	Radix gentianae	*Gentiana manshurica* Kitag. or *Gentiana scabra* var. *buergeri* (Miq.) Maxim. ex Franch. and Sav. or *Gentiana triflora* Pall. or *Gentiana rigescens* Franch	Clear heat and dry dampness, removing fire from the liver and gall bladder	Reserpine-induced mice	Downregulation of GluN2B receptors in the amygdala	Liu et al. (2014)

### 4.2 By regulating glutamatergic transmitter levels

Glutamate is the predominant excitatory neurotransmitter in the central nervous system and is involved in neuronal excitability, synaptic plasticity, and neurogenesis by activating various receptors, such as N-methyl-D-aspartic acid (NMDA) receptors, AMPA receptors, and metabotropic glutamate receptors (Orrego and Villanueva, 1993). Glutamate (Glu) and a small amount of another inhibitory amino acid neurotransmitter, gamma-aminobutyric acid (GABA), synergistically maintain the balance of excitatory/inhibitory circuits (Douglas and Martin, 2007; Sanacora et al., 2012). However, the balance of excitatory and inhibitory circuits has been found to be characteristically disturbed in MDD, mainly by dysfunction of the glutamatergic system, dysregulation of glutamatergic clearance, and metabolic regulatory mechanisms, which cause persistent glutamate accumulation. Structural and functional changes in regions related to mood and cognition and reduced synaptic activity are caused by excitotoxicity (e.g., excessive stimulation of glutamate receptors), which may lead to core symptoms of MDD, such as depressed mood, anhedonia, and cognitive dysfunction (Patel et al., 2015).

Glial glutamate transporter protein-1 (GLT-1) is one of the major Na-driven glutamate transporter proteins and plays an important role in maintaining control of synaptic glutamate concentration, and dysregulated expression of GLT-1 induces depressive symptoms (Weng et al., 2014). Schisantherin B is a lignan substance isolated from the traditional Chinese medicine Schisandra chinensis (Turcz.). Recent studies have shown that a single dose of Schisantherin B (15 mg/kg/d) can increase GLT-1 levels and alleviate symptoms of FST-induced depression in FST-induced depressed mice by promoting PI3K/AKT/mTOR pathways. Excessive accumulation of glutamate in brain tissues is a direct manifestation of the glutamate hypothesis of depression (Weng et al., 2014), so inhibiting excessive elevation of glutamate levels in the brain may be one of the approaches to treating depression with traditional Chinese medicines. Saikosaponin D and Gentiopicroside are the active ingredients in Chai Hu and Gentian, respectively, in which Saikosaponin D (1.50 mg/kg/d, 0.75 mg/kg/d) reduced glutamate levels in the CA1 region of the hippocampus of CUMS-exposed rats by modulating the Homer1-mGluR5 and downstream mTOR signaling pathways, and improved the behavioral performance of the rats in behavioral tests, proving its antidepressant effect (Liu et al., 2022). In contrast, Gentiopicroside (50 mg/kg/d, 100 mg/kg/d, 200 mg/kg/d) significantly reduced the immobility time during the forced swim test and increased the time in the center area in rifampicin-induced pain/depression dichotomy mice and the open-field test by down-regulating the GluN2B receptor in the amygdala, total distance traveled (Liu et al., 2014). More antidepressant mechanisms of herbal active ingredients by modulating glutamate transmitter levels are shown in Table 2.

**TABLE 2:** Antidepressant mechanisms of Chinese herbal medicine active ingredients regulating glutamate levels.

Chemical compound		Source Chinese medicine	Source plant		Traditional efficacy		Pharmacological model	Machine	Reference
Ginsenoside Rg1		Radix Ginseng	*Panax ginseng* C.A.Mey		Greatly tonify the original qi, restore the pulse and stabilize the body, tonify the spleen and benefit the lungs, engender fluid and nourish blood, calm the nerves and increase intelligence		Primary astrocytes	Reversal of aberrant activation of Cx43 phosphorylation and CORT-induced dysfunction of hemichannels, GJC and glutamatergic systems	Zhang et al. (2022a)
Schisantherin B		The fruit of Chinese magnolia vine	. *Schisandra chinensis* (Turcz.) Baill		Astringent and astringent, invigorating Qi and promoting the production of body fluid, tonifying the kidney and tranquilizing the heart		FST-induced depression in mice	Increasing glial glutamate transporter protein-1 levels by promoting the PI3K/AKT/mTOR pathway	Xu et al. (2019)
Saikosaponin D		Radix bupleuri	*Bupleurum chinense* DC. or *Bupleurum scorzonerifolium* Willd		Harmonize and release the exterior and interior, soothe the liver and resolve constraint, invigorating YING QI		CUMS rat	Suppresses neuroinflammation and oxidative stress; reduces glutamate levels in the hippocampus	Liu et al. (2022)
Gentiopicroside		Radix gentianae	*Gentiana manshurica* Kitag. or *Gentiana scabra* var. *buergeri* (Miq.) Maxim. ex Franch. and Sav. or *Gentiana triflora* Pall. or *Gentiana rigescens* Franch		Clear heat and dry dampness, removing fire from the liver and gall bladder		Reserpine-induced mice	Downregulation of GluN2B receptors in the amygdala	Liu et al. (2014)
Chiisanoside		Radix et Caulis Acanthopanacis Senticosi	*Acanthopanax senticosus* (Rupr.etMaxim.)Harms		Replenishing qi and fortify the spleen, tonify the kidney and calm the nerves		LPS-induced depression model in mice	Increased DA and GABA levels in the brain, decreased serum IL-6 and TNF-α levels, increased BDN levels	Bian et al. (2018)
Hyperforin		Hypericum perforatum	*Hypericum perforatum* L		Clear heat and resolve toxins, astringing to arrest bleeding, drain dampness		CUMS mice	Inhibition of glutamate uptake	Wonnemann et al. (2000)

In summary, abnormalities in the glutamatergic system are one of the recognized hypotheses in the pathogenesis of depression. Although there are fewer studies on the antidepressant effects of Chinese herbal medicines through glutamate, there is indeed some evidence of the effects of the active ingredients of Chinese herbal medicines on the glutamatergic system in the depressive process. This calls for more research to find the exact mechanism in order to develop new approaches and drugs in the treatment of depression.

### 4.3 By improving HPA axis dysfunction

**FIGURE 3:**
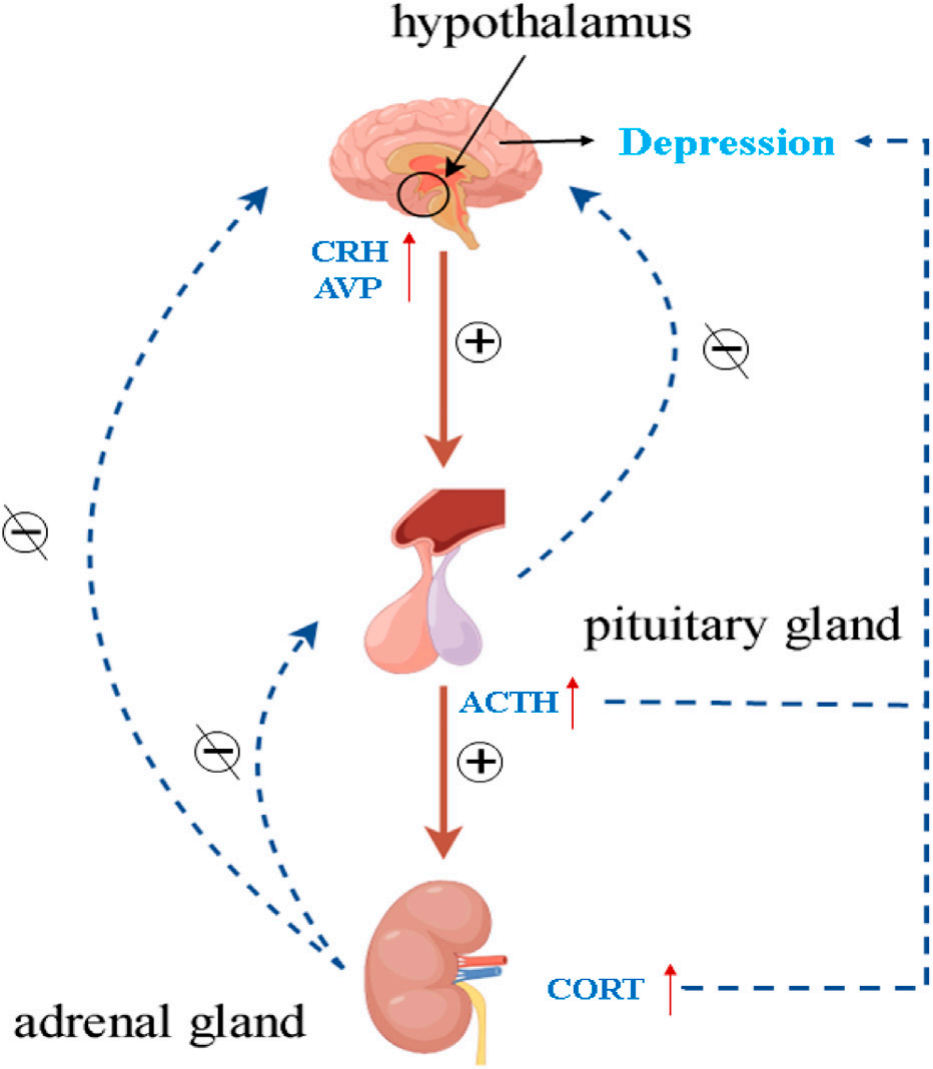
Dysregulation of the HPA axis promotes depression.

Various mood and cognitive disorders have been associated with the pathophysiology of the HPA axis. The HPA axis may be hyperactive in MDD patients, as studies have demonstrated (Keller et al., 2017). The regulation of the HPA axis primarily occurs through the influence of the hippocampus, which governs the secretion of corticotropin-releasing hormone (CRH) and arginine vasopressin (AVP) from the hypothalamic paraventricular nucleus (PVN). The anterior pituitary is stimulated by CRH to produce adrenocorticotropic hormone (ACTH), which then prompts the adrenal cortex to generate and release glucocorticoids (cortisol in humans and corticosterone in rodents) into the blood. Exogenous or endogenous stress may impair certain functions in the HPA axis, such as glucocorticoid resistance, i.e., dysfunction of the glucocorticoid receptor (GR), which impairs the negative feedback it mediates, and cause the pituitary gland and adrenal glands to enlarge, resulting in the disruption of the overstimulated HPA axis and increased levels of cortisol (CORT) in patients with depression (Figure 3) (Anacker et al., 2011; Nikkheslat et al., 2020). Therefore, the search for active ingredients of herbal medicines that can improve hippocampus-pituitary-adrenal axis dysfunction is a focus that requires urgent attention. Encouragingly, a large number of studies have been reported to elucidate the active ingredients of herbal medicines to alleviate depression by modulating the HPA axis, as well as their mechanisms of action.

Icariin, a flavonoid constituent isolated from Epimedium, was investigated in a chronic social defeat stress (CSDS)-induced mouse model of depression. The results showed that oral administration of 25 mg/kg and 50 mg/kg of Icariin reversed the CSDS-induced reduction in the duration of social interactions and attenuated the downregulation of serum GR and the increase in corticosterone levels in mice. The overexpression of CRH and ACTH as well as chronically elevated serum levels of CORT may contribute to the depressive-like behaviors (Vincent and Jacobson, 2014). Salidroside is a key active ingredient isolated from Rhodiola rosea. Salidroside, a key active ingredient isolated from Rhodiola rosea, was studied in a rat model of olfactory bulbectomy-induced depression. The results showed that oral administration of Salidroside (20 mg/kg/d/d, 40 mg/kg/d/d) significantly shortened the immobilization time of FST and TST and significantly increased the expression of GR and brain-derived neurotrophic factor (BDNF). In addition, Salidroside attenuated the expression of hypothalamic CRH and significantly reduced serum corticosterone levels. Suggesting that the antidepressant activity possessed by Salidroside may be mediated primarily by ameliorating abnormal HPA axis function (Yang et al., 2014). Rats were and exposed to a series of unpredictable mild stressors to establish a CUMS depression model, and after 19 days of gavage administration of Puerarin (a flavonoid glycoside extracted from the traditional Chinese medicine Pueraria lobata), it was found that concentrations of 60 and 120 mg/kg/d/d of Puerarin blocked the increase in serum CRH, CORT, and ACTH, implying that Puerarin’s antidepressant mechanism may be related to the regulation of HPA axis function (Qiu et al., 2017). The antidepressant mechanisms of the active ingredients of Chinese medicines by alleviating the dysfunction of the hypothalamic-pituitary-adrenal (HPA) axis are shown in Table 3.

**TABLE 3:** Antidepressant mechanism of improving HPA axis dysfunction by Chinese herbal active ingredients.

Chemical compound	Source Chinese medicine	Source plant	Traditional efficacy	Pharmacological model	Machine	Reference
Salidroside	Radix rhodiolae	*Rhodiola crenulata* (Hook. f. et Thoms.) H. Ohba (R. crenulata)	Clear the meridian and alleviate asthma, boost qi and invigorate blood	olfactory bulbectomy (OB) rat	Increased GR and BDNF expression in the hippocampus and decreased levels of CRH and serum corticosteron	Yang et al. (2014)
Emodin	Radix et rhizoma rhei	*Rheum palmatum* L. or *Rheum tanguticum* Maxim. ex Balf. or *Rheum officinale* Baill	Remove accumulation with purgation, heat-clearing and fire-purging, removing pathogenic heat from blood and toxicsubstance from the body	CUMS mice	Decreased plasma corticosterone concentration and increased BDNF expression levels	Li et al. (2014)
Puerarin	Radix puerariae	*Pueraria lobata* (Willd.)Ohwi	Expelling pathogenic factors from muscles for reducing heat, promoting fluid production to quench thirst, promoting eruption, dredging channels and activating collaterals	CUMS rat	Increased levels of 5-HT and 5-HIAA, downregulated levels of CRH, CORT, ACTH.	Qiu et al. (2017)
Magnolol	Cortex Magnoliae Officinalis	*Magnolia officinalis* Rehder and E.H.Wilson or *Magnolia officinalis* var. *biloba* Rehder and E.H.Wilson	Dry dampness to resolve phlegm, expel Qi and eliminate flatulence	CUMS rat	Decreased serum levels of CRH, ACTH and CORT and increased BDNF expression in the hippocampus	Wang et al. (2018)
ginsenoside Rg3	Radix Ginseng	*Panax ginseng* C.A.Mey	Greatly tonify the original qi, restore the pulse and stabilize the body, tonify the spleen and benefit the lungs, engender fluid and nourish blood, calm the nerves and increase intelligence	CUMS rat	Reduced CRH, corticosterone and ACTH levels	Qiu et al. (2017)
Curcumin	Rhizoma Curcumae Longae	*Curcuma Longa* L	Removing blood stasis and move qi, inducing menstruation to relieve menalgia	CUMS rat	Adjustment of the HPA axis	Wu et al. (2021)
cumuncis total alkaloids	Ramulus uncariae cumuncis	*Uncaria rhynchophylla* (Miq.)Miq. ex Havil	Extinguish wind and arresting convulsion, clear heat to suppressing the hyperactive liver	chronic restraint stress (CRS) mice	Reduced serum levels of CORT, TNF-α, and NO.	(Liu et al., 2017)
Icariin	Herb of Shorthorned Epimedium	*Epimedium brevicornu* Maxim	Tonifying kidney-yang, strengthening muscles and bones, dispelling wind-dampness	CSDS mice	Attenuates social failure-induced downward GR adjustments	Wu et al. (2011)

### 4.4 By regulating neurotrophic factor levels

Neuroplasticity, the process of growth and adaptation at the neuronal level, plays a crucial role in the modification of neurogenesis and the morphology of mature neurons. However, restricted neurogenesis and disturbances in neuronal morphology can contribute to the development of MDD-like symptoms (Castrén, 2013; Allen and Lyons, 2018). Neuroplasticity, including synaptic plasticity, neurotransmission, neuronal survival, growth, and differentiation, is regulated by a combination of neurotrophic factors such as BDNF, glial cell line-derived neurotrophic factor (GDNF), vascular endothelial growth factor (VEGF), and nerve growth factor (NGF) (Song et al., 2017). These factors work synergistically to enhance and support neuroplasticity processes (Thoenen, 1995; Wang et al., 2022). Therefore, modulation of neurotrophic factor levels may be the direction of herbal medicine to improve depression.

There has been a large body of evidence demonstrating the effects of active ingredients in herbal medicines on the levels of neurotrophic factors. For example, resveratrol, a natural polyphenol, was found to improve CUMS-induced depressive-like behavior and cognitive deficits in mice by activating the Sirt1/miR-134 pathway and upregulating downstream cAMP response element-binding protein (CREB)/BDNF levels in the hippocampus (Shen et al., 2018). Long-term administration of ginsenoside Rg1 (40 mg/kg/d for 5 weeks) significantly ameliorated neuronal structural abnormalities and biochemical changes induced by CUMS stress and prevented depressive-like behaviors in rats with CUMS, which may be based on the mechanism of neuroprotective and antidepressant-like effects exhibited through activation of the CREB-BDNF system in the basolateral amygdala (Zhu et al., 2016). Ethanolic extracts of Dendrobium flowers increased the expression of NGF and BDNF at both transcriptional and protein levels, decreased immobilization time and increased sucrose consumption in a forced swimming test in mice (Zhu et al., 2021). It suggests that the antidepressant effects of the Dendrobium flowers may be related to the modulation of neurotrophic factor levels. Table 4 exhibits other Central Asian active ingredients and extracts that target neurotrophic factors.

**TABLE 4:** Antidepressant mechanism of Chinese herbal medicine active ingredients regulating the level of neurotrophic factor.

Chemical compound	Source Chinese medicine	Source plant	Traditional efficacy	Pharmacological model	Machine	Reference
Salidroside	Radix rhodiolae	*Rhodiola crenulata* (Hook. f. et Thoms.) H. Ohba (R. crenulata)	Clear the meridian and alleviate asthma, boost qi and invigorate blood	OB rat	Increased GR and BDNF expression in the hippocampus and decreased levels of CRH and serum corticosteron	Yang et al. (2014)
Macranthol	Illicium verum	*Illicium dunnianum* Tutcher	Dissipating blood stasis for subsidence ofswelling, dispel wind and overcome dampness, relieve pain	CUMS mice	Improvement of hippocampal BDNF levels	Luo et al. (2015)
Hyperforin	Hypericum perforatum	*Hypericum perforatum* L	Clear heat and resolve toxins, astringing to arrest bleeding, drain dampness	CUMS mice	Regulates BDNF and zinc levels	Szewczyk et al. (2019)
Huperzine A	All-grass of Snakefoot clubmoss	*Huperzia serrata* (Thunb. ex Murray) Trevis	Clear heat and resolve toxins, promote tissue regeneration and close wound and stanch bleeding, dissipate (blood) stasis	PSD rat	Upregulation of hippocampal CREB and BDNF levels	Du et al. (2017)
Cucurbitacin IIA	Radix hemsleyae	*Hemsleya amabilis* Diels. or *Hemsleya macrosper*ma C.Y.Wu	Clear heat and resolve toxins, invigorate the stomach and relieve pain	CUMS mice	Elevated levels of BDNF, CREB in the amygdala	Zhou et al. (2017)
Emodin	Radix et rhizoma rhei	*Rheum palmatum* L. or *Rheum tanguticum* Maxim. ex Balf. or *Rheum officinale* Baill	Remove accumulation with purgation, heat-clearing and fire-purging, removing pathogenic heat from blood and toxicsubstance from the body	CUMS mice	Decreased plasma corticosterone concentration and increased BDNF expression levels	Li et al. (2014)
Peony total glycosides	Cortex moutan	*Paeonia suffruticosa* Andrews	Clear heat and cool the blood, activate blood and resolve stasis	CORT-induced rats	Increased brain BDNF levels	Mao et al. (2012)
Geniposide	Fructus gardeniae	*Gardenia jasminoides* J.Ellis	Eliminating fire and remove vexation, clear heat and drain dampness, removing pathogenic heat from blood and toxicsubstance from the body	CUMS mice	Inhibition of inflammatory cytokine production and upregulation of BDNF levels	Chen et al. (2021)
Gastrodin	Rhizoma gastrodiae	*Gastrodia elata* Blume	Relieving spasm by subduing liver-wind, pacify the liver to subdue yang, dispel wind to free the collateral vessels	CUMS rat	Improvement of hippocampal BDNF levels	Zhang et al. (2014)
Paeoniflorin	Radix Paeoniae Alba	*Paeonia lactiflora* Pall	Nourish blood to regulate menstruation, astringing YIN to stop sweating, easing the affected liver to relieve pain, pacify the liver to subdue yang	CUMS mice	Enhancement of BDNF expression in the hippocampus	Liu et al. (2019b)
Magnolol	Cortex Magnoliae Officinalis	*Magnolia officinalis* Rehder and E.H.Wilson or *Magnolia officinalis* var. *biloba* Rehder and E.H.Wilson	Dry dampness to resolve phlegm, expel Qi and eliminate flatulence	CUMS rat	Increased BDNF expression in the hippocampus	Wang et al. (2018)
Ginsenoside Rg1	Radix Ginseng	*Panax ginseng* C.A.Mey	Greatly tonify the original qi, restore the pulse and stabilize the body, tonify the spleen and benefit the lungs, engender fluid and nourish blood, calm the nerves and increase intelligence	CUMS rat	Increased BDNF expression in the hippocampus	Zhu et al. (2016)
Chiisanoside	Radix et Caulis Acanthopanacis Senticosi	*Acanthopanax senticosus* (Rupr.etMaxim.)Harms	Replenishing qi and fortify the spleen, tonify the kidney and calm the nerves	LPS-induced depression model in mice	Increased DA and GABA levels in the brain, decreased serum IL-6 and TNF-α levels, increased BDN levels	Bian et al. (2018)
Fuzi polysaccharide-1	Radix Aconiti Lateralis Preparata	*Aconitum carmichaelii* Debeaux	Restoring yang to save from collapse, supplement fire and assist yang, dispersing cold for relieving pain	CSDS mice	Increased BDNF levels in the hippocampus	Yan et al. (2010)
Dendrobii Caulis Officinale ethanol Extract	Dendrobii Caulis	*Dendrobium nobile* Lindl	Promoting the production of body fluid and stomach, clearing heat and nourishing Yin	CUMS mice	Increased expression of NGF and BDNF through a cAMP-dependent mechanism	Zhu et al. (2021)
tetrahydrocurcumin	Rhizoma Curcumae Longae	*Curcuma Longa* L	Removing blood stasis and move qi, inducing menstruation to relieve menalgia	CRS mice	Enhanced expression of BDNF and GDNF	Yang et al. (2023)
Resveratrol	giant knotweed rhizome	*Reynoutria japonica* Houtt	Dispelling wind, inducing diuresis, breaking up blood stasis and promoting menstruation	CUMS mice	Activation of the Sirt1/miR-134 pathway upregulates downstream CREB/BDNF levels in the hippocampus	Shen et al. (2018)
*Ocimum sanctum L* ethanol extract	Sweet Basil herb	*Ocimum sanctum L*	Pain reliever, asthma reliever	OB mice	Reduced expression levels of genes encoding VEGF and VEGF receptor type 2 (VEGFR2)	Le et al. (2021)
*Allium macrostemon* Bunge aqueous extract	*Allium macrostemon* Bunge	*Allium macrostemon* Bge.*or Allium chinensis* G. Don	Promoting Yang and dispersing knots, moving Qi and directing stagnation	FST and TST mice	Promoting neurogenesis and BDNF expression levels	Wu et al. (2023)

### 4.5 By improving inflammation

In the past few decades, a growing body of evidence has highlighted the involvement of systemic immune activation and inflammatory processes in the development of MDD (Maes, 1999; Gibney and Drexhage, 2013). Inflammation, which arises from the activation of the immune system, typically manifests as a localized response triggered by irritation, injury, or infection. However, when dysregulated, immune cells and mechanisms within the body can contribute to the onset of diseases like MDD (Murphy et al., 2007). There is evidence that patients with depression have elevated levels of inflammatory cytokines such as tumor necrosis factor alpha (TNF-α), interleukin 1β (IL-1β), and interleukin 6 (IL-6) (Beurel et al., 2020). This may indicate the presence of inflammatory processes in patients with MDD.

Using LPS-induced depressed mice, after gavage administration of Chiisanoside (5.0 mg/kg/d), it effectively reduced serum IL-6 and TNF-α levels, as well as improved oxidative stress-related indices, and significantly increased immobility time of TST and FST, suggesting that Chiisanoside may improve depression by modulating the levels of inflammatory factors. Under stress, NF-κB is activated to promote the production of IL-1β proximate (Bian et al., 2018). Fan et al. (2018) found that pretreatment with Curcumin (40 mg/kg/d) for 5 weeks ameliorated the depressive-like effects and suppressed the inflammatory response and neuronal structural abnormalities in CUMS-exposed mice, with a mechanism of action similar to that of IL-1β or NF-κB antagonists. There is evidence that High Mobility Group Box 1 (HMGB1) can play an important role in neurodegenerative diseases by mediating neuroinflammation (Chi et al., 2015). Icariin was found to promote HMGB1 translocation to the nucleus and, on the one hand, inhibit HMGB1-RAGE anti-inflammatory signaling and, on the other hand, may activate TLR4-NF-κB signaling and increase p65 expression in the nucleus to promote nerve regeneration, effectively improving social avoidance behavior in the social interaction test and time spent in the central area in the open field test in rats (Liu L. et al., 2019). Other studies involving the improvement of immune and inflammatory dysregulation mechanisms are shown in Table 5.

**TABLE 5:** Inflammation inhibition and antidepressant mechanism of Chinese herbal active ingredients.

Chemical compound	Source Chinese medicine	Source plant	Traditional efficacy	Pharmacological model	Machine	Reference
Ginkgolide	Ginkgo	*Ginkgo biloba* L	Activate blood and resolve stasis, dredging collaterals and relieving pain, astringing lung QI for relieving asthma	Depressed mice after myocardial infarction	Regulation of IL-1β, 5-HT and DA levels	Ge et al. (2020)
Saikosaponin D	Radix bupleuri	*Bupleurum chinense* DC. or *Bupleurum scorzonerifolium* Willd	Harmonize and release the exterior and interior, soothe the liver and resolve constraint, invigorating YING QI	CUMS rat	Suppresses neuroinflammation and oxidative stress	Wang et al. (2021)
Geniposide	Fructus gardeniae	*Gardenia jasminoides* J.Ellis	Eliminating fire and remove vexation, clear heat and drain dampness, removing pathogenic heat from blood and toxicsubstance from the body	CUMS mice	Inhibition of inflammatory cytokine production and upregulation of BDNF levels	Chen et al. (2021)
Gastrodin	Rhizoma gastrodiae	*Gastrodia elata* Blume	Relieving spasm by subduing liver-wind, pacify the liver to subdue yang, dispel wind to free the collateral vessels	CUMS rat	Decreased IL-1β expression levels	Wang et al. (2014)
ginsenoside Rg3	Radix Ginseng	*Panax ginseng* C.A.Mey	Greatly tonify the original qi, restore the pulse and stabilize the body, tonify the spleen and benefit the lungs, engender fluid and nourish blood, calm the nerves and increase intelligence	LPS-induced depression in mice	Reduces plasma levels of IL-6 and TNF-α	Kang et al. (2017)
Chiisanoside	Radix et Caulis Acanthopanacis Senticosi	*Acanthopanax senticosus* (Rupr.etMaxim.)Harms	Replenishing qi and fortify the spleen, tonify the kidney and calm the nerves	LPS-induced depression model in mice	Increased DA and GABA levels in the brain, decreased serum IL-6 and TNF-α levels, increased BDN levels	Bian et al. (2018)
cumuncis total alkaloids	Ramulus uncariae cumuncis	*Uncaria rhynchophylla*(Miq.)Miq. ex Havil	Extinguish wind and arresting convulsion, clear heat to suppressing the hyperactive liver	CRS mice	Reduced serum levels of CORT, TNF-α, and NO.	Liu et al. (2017)
Icariin	Herb of Shorthorned Epimedium	*Epimedium brevicornu* Maxim	Tonifying kidney-yang, strengthening muscles and bones, dispelling wind-dampness	CUMS mice	Inhibition of HMGB1-RAGE signaling and simultaneous activation of TLR4-NF-κB signaling	Liu et al. (2019a)
Curcumin	Rhizoma Curcumae Longae	*Curcuma Longa* L	Removing blood stasis and move qi, inducing menstruation to relieve menalgia	CUMS rat	Reduction of serum inflammatory factor levels ameliorates the dysregulation of neuronal structural plasticity induced by the IL-1β/NF-κB pathway	Fan et al. (2018)
Paeonia lactiflora Pall. Polysaccharide	White Peony Root	*Paeonia lactiflora* Pall	Nourishing Blood, softening the Liver, easing pain in the middle, astringing Yin and collecting sweat	CUMS mice	Reduced IL-1β, IL-6, and TNF-α levels and increased 5-HT levels in CUMS mice	Zhou et al. (2023)

### 4.6 By regulating the intestinal flora

The human gut flora is a diverse and complex ecosystem consisting of trillions of microorganisms, including bacteria, viruses, archaea, and fungi (Kc et al., 2020), that have a role in human health. Gut flora participate in a bidirectional communication pathway with the central nervous system (CNS), known as the microbiota-gut-brain axis (Dinan and Cryan, 2017; Chang et al., 2022). In contrast, dysbiosis disrupts the brain-gut-microbiota axis, induces a neuroimmune inflammatory response, disrupts the function of the intestinal mucosa and the blood-brain barrier, and directly stimulates the vagus nerve, enteric nervous system, spinal nerves, and HPA axis. These interactions have been implicated in the development of neurological disorders, including MDD (Tang and Cao, 2021).

As the link between gut flora and the onset of depression becomes more deeply understood, more and more studies are finding that herbs can improve depression by regulating gut flora. Patchouli essential oil, the main active ingredient in patchouli, is used in aromatherapy for stress relief. Oral administration of patchouli essential oil (0.8 mL/kg) significantly attenuated the depression-like behavior induced by CUMS stress in mice in FST. It was found to increase the relative abundance of several probiotics (including *Bacteroides* and Blautia) and improve the levels of metabolites short-chain fatty acids (SCFAs) and restore hippocampal 5-HT levels regarding the gut microbiota as analyzed by 16S rRNA gene sequencing (Ouyang et al., 2024). In addition, changes in gut flora affect the immune system and generate an inflammatory response, which in turn induces or exacerbates the depressive response (Peirce and Alviña, 2019). The establishment of a CUMS rat model of depression significantly increased the abundance of beneficial bacteria (*Lactobacillus* and Oscillospira) in the rat gut after the intervention of administering a certain amount of Astragaloside IV, as well as modulating the imbalance of Th17/Treg cells and the abnormal levels of anti-inflammatory and pro-inflammatory factors. This resulted in improved depression-like behavior in rats, as evidenced by weight gain, upregulation of sucrose preference, and decreased immobility time. This implies that Astragaloside IV is able to improve the inflammatory response by regulating intestinal flora, which in turn reduces depressive symptoms (Liu et al., 2024). We have compiled a list of active ingredients of some Chinese herbal medicines (Table 6), which have antidepressant effects by modulating the intestinal flora.

**TABLE 6:** Antidepressant mechanism of Chinese herbal medicine active ingredients regulating intestinal flora.

Chemical compound	Source Chinese medicine	Source plant	Traditional efficacy	Pharmacological model	Machine	Reference
Hyperforin	Hypericum perforatum	*Hypericum perforatum* L	Clear heat and resolve toxins, astringing to arrest bleeding, drain dampness	CUMS mice	Regulates intestinal flora	Zhang et al. (2022b)
Icariin	Herb of Shorthorned Epimedium	*Epimedium brevicornu* Maxim	Tonifying kidney-yang, strengthening muscles and bones, dispelling wind-dampness	A model of prenatal stress-induced depression in rat pups	Improves metabolism and increases the abundance of probiotics in the gut	Dong et al. (2024)
Pogostemon cablin essential oil	Cablin Patchouli Herb	*Pogostemon cablin* (Blanco) Benth	Aromatizing and transforming dampness, harmonizing the stomach and stopping vomiting, dispelling summer heat and relieving symptoms	CUMS rat	Increased relative abundance of Synechocystis and cyanobacteria, decreased relative abundance of Ruminococcus_1 and Ruminococcus_2, and regulated short-chain fatty acid levels	Ouyang et al. (2024)
Astragaloside IV	Radix Astragali	*Astragalus membranaceus* (Fisch.) Bunge *or Astragalus mongholicus* Bunge	Tonifying Qi and consolidating the surface, diuretic and detoxifying, draining pus, astringing sores and regenerating muscles	CUMS rat	Increased abundance of beneficial bacteria (*Lactobacillus* and Oscillospira), significant modulation of Th17/Treg cell imbalance, and abnormal levels of anti-inflammatory and pro-inflammatory factors	Liu et al. (2024)
Cryptotanshinone	Danshen Root	*Salvia miltiorrhiza* Bunge	Removing blood stasis and relieving pain, invigorating blood circulation and promoting menstruation, clearing the mind and removing vexation	CUMS rat	Attenuates harmful bacterial changes associated with depression, reduces inflammatory factor levels, and modulates the PI3K-AKT pathway to exert its effects	Bian et al. (2023)
Polygalae Radix Oligosaccharide Esters	Radix Polygalae	*Polygala tenuifolia* Willd. or *Polygala sibirica* L	Calm the nerves and increase intelligence, coordinate the heart and kidney, dispel phlegm, disperse swelling	CUMS rat	Regulating the imbalance of intestinal flora in rats, regulating the levels of SCFAs in feces as well as serum LPS and IL-6 levels	Chen et al. (2023)
Paeonia lactiflora Pall. Polysaccharide	White Peony Root	*Paeonia lactiflora* Pall	Nourishing Blood, softening the Liver, easing pain in the middle, astringing Yin and collecting sweat	CUMS mice	Reduced IL-1β, IL-6, and TNF-α levels and increased 5-HT levels in CUMS mice	Zhou et al. (2023)
Matrine	Lightyellow Sophora Root	*Sophora flavescens* Aiton	Clearing heat, drying dampness and killing parasites	CUMS mice	Regulates disorders of intestinal flora and metabolites, reduces levels of pro-inflammatory cytokines in peripheral blood circulation and brain regions, and increases levels of BDNF in the brain	Zhang et al. (2023b)

In summary, intestinal flora-associated depression has received much attention in recent years. As the interactions and relationships between changes in intestinal flora and depression become clearer and clearer, the mechanism of antidepressant effects of traditional Chinese medicines by regulating intestinal flora will continue to be revealed.

## 5 Application of classical and famous Chinese medicine formulas in antidepressants

Depression has a complex and diverse etiology and pathogenesis, and single-targeted therapeutic strategies with traditional antidepressants may be ineffective. In contrast, Chinese herbal formulas, with their systemic properties and multi-targeting characteristics, may provide advantages for the treatment and prevention of depression. In traditional Chinese medicine, Chinese herbal formulas are commonly used drug therapies, consisting of a combination of multiple Chinese medicines, providing a material basis for the diversity of antidepressant mechanisms. They can adapt to the characteristics of the diversity of causative factors and the complexity of lesions in depression through the integration of the regulating effects of multi-link, multi-level, and multi-targets. At the same time, different Chinese medicines can synergistically enhance antidepressant efficacy through mutual promotion and enhancement (Zhuang et al., 2023).

In the records of traditional Chinese medicine, many Chinese herbal formulas have been proposed and applied to treat depression (“yu-shen” in Chinese medicine theory). Some of the classical formulas are hundreds or even thousands of years old and are still used in current clinical practice. In this paper, we summarize eight classic TCM formulas (Table 7), which have been shown to have multiple mechanisms of action and targets. Among them, Xiaoyao San (XYS), Chaihu Shuogan San (CHSGS), and Kaixin San (KXS) have received strong attention for their remarkable efficacy. We will focus on a systematic review of these three classic prescriptions.

**TABLE 7:** Antidepressant studies of classical formulas.

Classical formulas	Prescription composition	*Source*	Pharmacological model	Dosages	Machine	Basis of the hypothesis	Reference
Xiaoyao San	Radix Bupleuri (chai hu), Radix Angelicae (dang gui), Radix Paeoniae Alba(bai shao), Rhizoma Atractylodis Macrocephalae (bai zhu), Poria (fu ling), Rhizoma Zingiberis Recens (sheng jiang), Herba Menthae (bo he), Radix et Rhizoma Glycyrrhizae Praeparata cum Melle (zhi gancao)	*TaiPingHuiMin HeJiJvFang*	CUMS rat	2.224 g/kg/d	Reducing glutamate levels in the CA1 region of the hippocampus	glutamatergic transmitter	Zhou et al. (2021)
			CRS rat	2.224 g/kg/d	Regulating the abundance of *Mycobacterium* anthropophilum, *Mycobacterium avium*, *Mycobacterium* thickum*etc.*	Intestinal flora	Zhu et al. (2019)
			CRS rat	200、400 and 600 mg/kg/d	Improvement of 5-HT and 5-HIAA levels in the cerebral cortex	Monoamine transmitte	Bao et al. (2008)
			CUMS rat	1.9 g/kg/d	Increased BDNF expression, downregulated CRH receptor 2 levels, and inhibited HPA axis hyperactivation	Neurotrophic factor; HPA Axis	Zhu et al. (2014)
			CUMS mice	0.658 g/kg/d	Amelioration of intestinal dysbiosis and inhibition of complement C3-mediated aberrant synaptic pruning in microglia	Intestinal flora	Hao et al. (2024)
Chaihu Shugan San	Radix Bupleuri (chai hu), Radix Paeoniae (sha oyao), Rhizoma Cyperi (Xiang fu), Fructus Aurantii (zhi qiao), Dried Tangerine Peel (chen pi), Rhizoma Chuanxiong (chuan xiong), Radix Glycyrrhizae (gan cao)	*JiangYueQuanShu*	Middle cerebral artery occlusion (MCAO)/CUMS rat	4.4 g/kg/d	Regulation of microglia polarization and inhibition of neuroinflammation	Inflammations	Fan et al. (2023)
			CUMS mice	20 mg/kg/d	Increasing the relative abundance of Bifidobacteria and serum levels of various bile acids	Intestinal flora	Ma et al. (2022)
			Depression and epilepsy in rats	2.7 g/kg/d	Promotes 5-HT1A receptor mRNA expression in the hippocampus	Monoamine transmitte	Yang et al. (2016)
			CRS mice	0.5、1 and 4 g/kg/d	Regulation of gut microbiota and modulation of NF-κB-mediated BDNF expression	Intestinal flora, neurotrophic factor	Han et al. (2021)
Kaixin San	Ginseng (ren shen), Radix Polygalae (yuan zhi), Rhizoma Acori Tatarinowii (shi chang pu), Poria (fu ling)	*BeiJiQianJinYaoFang*	CUMS mice	3 and 10 g/kg/d	Reduced expression of pro-inflammatory cytokines such as IL-1β, IL-2 and TNF-α in the hippocampus	Inflammations	Qu et al. (2021)
			CMS rat	60.9 、182.7 and 548.1 mg/kg/d	Restoration of monoamine neurotransmitter levels in the brain and neurotrophic factor levels in the cortex	Monoamine transmitte; neurotrophic factor	Yan et al. (2016), Zhu et al. (2017)
			CUMS mice	1.5 g/kg/d	Upregulation of NGF, BDNF and Trkb receptor expression in the hippocampus	Neurotrophic factor	Zhu et al. (2017)
			CUMS mice	3 and 10 g/kg/d	Improvement of small intestinal microbiota composition, reduction of LPS and pro-inflammatory cytokines	Intestinal flora	Cao et al. (2020)
Banxia HoupoTang	Rhizoma Pinelliae (ban xia), Magnolia bark (hou po), Poria (fu ling), Rhizoma Zingiberis Recens (sheng jiang), Folium Perillae (su ye)	*JinKuiYaoLue*	CUMS rat	3.29 and 6.58 g/kg/d	Inhibition of NLRP3 inflammatory vesicle activation	Inflammations	Jia et al. (2017)
			CMS rat	130 mg/kg/d	Increases 5-HT, 5-HIAA levels and decreases IL-2 levels	Monoamine transmitte; inflammations	Li et al. (2003)
Baihe Dihaung Tang	Bulbus Lilii (bai he), Radix Rehmanniae Recens (sheng di huang)	*JinKuiYaoLue*	CUMS rat	3.2 and 12.6 g/kg/d	Decreased IL-1β levels, increased 5-HT expression	Inflammations; monoamine transmitte	(Zhou et al., 2018)
			CUMS mice	6 and 24 g/kg/d	Reduces serum concentrations of CORT, ACTH.	HPA Axis	(Guan et al., 2013)
Sini San	Radix Bupleuri (chai hu), Radix Paeoniae Alba(bai shao), Fructus Aurantii Immaturus (zhi shi), Radix et Rhizoma Glycyrrhizae Praeparata cum Melle (zhi gan cao)	*ShangHanLun*	CUMS rat	2.5 and 5 g/kg/d	Reduction of hippocampal tissue IL-18, IL-1β levels and inhibition of NLRP3 inflammatory vesicle expression	Inflammations	(Wang et al., 2022)
			CUMS rat	2.5, 5 and 10 g/kg/d	Upregulation of 5-HTAA, p-CREB and BDNF expression in hippocampus	Monoamine transmitte; neurotrophic factor	Cao et al. (2019a)
Yueju Pill	Rhizoma Cyperi (Xiang fu), Rhizoma Chuanxiong (chuan xiong), Fructus Gardeniae (zhi zi), Rhizoma Atractylodis (cang zhu), Liushen Qv (liu shen qv)	*DanXiXinFa*	LPS-induced depression model in mice	2 g/kg/d	Reduced serum levels of IL-1β, TNF -α, and IL -10, and increased BDNF and TrkB expression in the hippocampus	Inflammations; neurotrophic factor	(Nie et al., 2020)
			CUMS rat	1.9, 3.8 and 7.6 g/kg/d	Increased levels of monoamine transmitters in the hippocampus and restoration of the structure and diversity of the intestinal flora	Monoamine transmitte; intestinal flora	Qu et al. (2024)
			CUMS mice	1.8 g/kg/d	Upregulation of ERK/AKT-mediated GLT-1 expression reduces Glu levels in hippocampal species	Glutamatergic transmitter	Zhang et al. (2023a)
Zuojin Pill	Rhizoma Coptidis (huang lian), Fructus Evodiae (wu zhu yv)	*DanXiXinFa*	CUMS mice	450 and 910 mg/kg/d	Inhibition of excess pro-inflammatory factors suppresses neuroinflammation *via* the SPOP/MyD88/NF-κB pathway	Inflammations	Tao et al. (2023)
			CUMS mice	225, 450 and 910 mg/kg/d	Increased expression of BDNF, TPH2 and 5-HT in the hippocampus	Monoamine transmitte	Wang et al. (2023)

### 5.1 XYS

XYS is a classic formula in TCM, which was first developed in the Song Dynasty (960–1127 A.D.) from the “TaiPingHuiMin HeJiJvFang” and has been used in the treatment of psychiatric disorders for thousands of years. XYS is made up of eight commonly used Chinese herbs: Chai Hu, Dang Gui, Bai Shao, Bai Zhu, Fu Ling, Sheng Jiang, Bo He, and Zhi Gan Cao. In recent years, a large number of studies have demonstrated that XYS can exert antidepressant effects through various mechanisms. Zhou et al. (2021) found that glutamate and serum CORT levels were abnormally high in the hippocampal CA1 region of CUMS-induced depressed rats, and that the administration of XYS (2.224 g/kg/d) significantly reduced glutamate and CORT levels, and improved the depression-like symptoms of the rats. Moreover, the antidepressant effect of XYS may be exerted through the NR2B and PI3K/Akt signaling pathway. In order to treat MDD, Zhu et al. (2019) explored the effects of XYS on depressive behaviors. A CRS rat model of MDD was established, and a 16S rDNA high-throughput method was used to sequence fecal specimens to detect the structure and changes of intestinal flora. The results showed that at the phylum level, XYS regulated the abundance of Bacteroidetes, Proteobacteria, Firmicutes, Chloroflexi, and Planctomycetes. At the genus level, XYS decreased the abundance of Prevotellaceae_Ga6A1_group, Prevotellaceae_UCG-001, and Desulfovibrio, and increased the abundance of Ruminococcaceae in order to improve depression-like behavior. It was also found that this process involves mechanisms that may be related to short-chain fatty acids, lipopolysaccharides, and intestinal inflammation. Recent studies have found that dysbiosis of gut flora can induce the development of depressive-like behaviors through abnormal synaptic pruning of microglia mediated by complement C3, and that XYS was able to inhibit abnormal synaptic pruning by regulating gut flora to improve depressive-like behaviors in CUMS mice (Hao et al., 2024). In addition, other studies have shown that XYS can also ameliorate MDD by modulating monoamine neurotransmitters, BDNF, and neuroendocrine levels (Bao et al., 2008; Zhu et al., 2019).

### 5.2 CHSGS

CHSGS was first introduced in the classic medical text “JingYueQuanShu” from the Ming Dynasty. It is composed of Chai Hu, Shao Yao, Xiang Fu, Zhi Ke, Chen Pi, Chuan Xiong, and Gan Cao. In Chinese medicine, it is used to relieve liver qi stagnation. Modern research on it has found that CHSGS is able to alleviate MDD by regulating intestinal flora, reducing inflammation, and increasing monoamine transmitter levels. For example, post-stroke MDD was modeled by subjecting male rats to middle cerebral artery occlusion and chronic unpredictable mild stress. CHSGS (4.4 g/kg/d) was found to modulate microglia polarization by activating the JAK/STAT3-GSK3β/PTEN/Akt pathway, suggesting that CHSGS can exert antidepressant effects by inhibiting neuroinflammation (Fan et al., 2023). Ma et al. (2022) transplanted gut microbes from CHSGS-treated mice into untreated CUMS mice, restored serum levels of hyocholic acid and 7-ketodeoxycholic acid in depressed mice, increased BDNF levels, and alleviated depression-like symptoms. The comorbidity of epilepsy and MDD is common in neuropsychiatry (Alhashimi et al., 2022). A rat model of depressive epilepsy was established by inducing epilepsy with chronic hairy fructosamine followed by exposure to chronic mild stress, and it was found that CHSGS (2.7 g/kg/d) increased the expression of 5-HT1A receptor mRNA and cell proliferation in the hippocampal dentate gyrus of depressive epileptic rats, which was effective in ameliorating the depressive symptoms (Yang et al., 2016).

### 5.3 KXS

KXS was initially described in the ancient Chinese book “BeiJiQianJinYaoFang” written by Sun Simiao in the Tang Dynasty around the year 652 AD. Initially used to treat dementia and forgetfulness, it is composed of ginseng, Yuan Zhi, shi chang pu, and fu ling. In traditional Chinese medicine theory, MDD is believed to be caused by qi, dampness, and phlegm, ultimately leading to brain dysfunction (Pei et al., 2020). KXS functions to tonify qi, nourish the heart, expel dampness, and resolve phlegm, thus aiding in the treatment of MDD. More and more research indicates that KXS can effectively treat MDD by regulating neurotrophic factors, neurotransmitters, gut microbiota, and inflammation (Fu et al., 2019). In a preclinical study using fluoxetine (7.2 mg/kg/d) as a positive control, administering KXS extract orally at doses of 3 or 10 g/kg/d significantly improved depressive-like behaviors in chronic unpredictable mild stress (CUMS) rats in sucrose preference, forced swimming, and tail suspension tests, thereby alleviating symptoms of anhedonia, despair, and anxiety. Additionally, KXS inhibits the activation of microglial cells and significantly reduces the expression of pro-inflammatory cytokines such as IL-1β, IL-2, and TNF-α in the mouse hippocampus by inhibiting the TLR4/IKK/NF-κB pathway (Qu et al., 2021). KXS also helps regulate neurotrophic factor metabolic pathways; after oral administration of KXS (1.5 g/kg/d) daily for several days, it significantly alleviates depressive symptoms in mice subjected to chronic unpredictable mild stress, as evidenced by increased sucrose consumption, reduced immobility time in forced swimming, and increased locomotor activity. It was found that KXS achieves this effect by upregulating the expression of NGF, BDNF, and Trkb receptors in the hippocampus, which was confirmed by treatment with respective inhibitors tPA-stop and K252a (Zhu et al., 2017).

## 6 Discussion

MDD’s pathogenesis is multifaceted and convoluted. Despite the fact that present studies have shed light on its possible pathogenesis from various angles, the extent to which each pathway contributes differs among individuals and still fails to explain the full range of pathogenic mechanisms. Based on this dilemma, new treatments are scarce, and the mechanisms studied are relatively homogeneous, lacking integration and systematic research. MDD is a disease with multiple pathways and factors, which requires in-depth study of the interactions among pathogenic mechanisms and exploration of the intrinsic linkages and molecular pathways through which various pathogenic factors affect each other, rather than just following the causal logic of the association.

With the development of TCM, TCM has received more and more attention due to its outstanding therapeutic effect and fewer side effects. TCM natural monomers are also supplements to traditional antidepressants to minimize adverse effects. These single components have higher biological activity and are more likely to enter cells to exert medicinal effects. They have advantages of fast onset of action and low side effects. In addition, as summarized in this paper, it was found that, compared with traditional synthetic drugs, many of the active ingredients of Chinese medicines do not have a single antidepressant mechanism and can achieve antidepressant effects through multiple pathways. For example, Icariin and Curcumin are able to act on MDD through a variety of mechanisms, such as neurotrophic factor, HPA axis, and inflammation. This may be an advantage of the natural active ingredients of herbal medicine instead of traditional antidepressant drugs. Overall, the natural active ingredients in TCM provide a promising direction for the development of novel antidepressant drugs.

Formulas are one of the most commonly used drug therapies in TCM, especially the classical formulas, which have been in use for a long time and have clear therapeutic effects. To this day, they are still widely used in clinical practice, and a range of herbal combinations provide the material basis for diverse antidepressant mechanisms. However, due to the complexity of the ingredients, current studies have mainly focused on their overall effects on MDD, and the exact relationship between the active ingredients in the formula and the pathogenesis of MDD has rarely been reported. It is also not clear how the multiple ingredients synergize with each other to treat MDD through different mechanisms. In addition, there are many traditional antidepressant formulas in China, and the antidepressant effects of some of them have not yet been fully explored, including many classical formulas that have been determined to have significant antidepressant effects but have been neglected due to a lack of research. Therefore, more comprehensive and in-depth studies are needed to elucidate the exact active ingredients in classical prescriptions and their molecular pharmacology related to the prevention and treatment of MDD.

In conclusion, this review summarizes the latest information and insights from research on the use of herbal medicines in the treatment of MDD. Much evidence suggests that the antidepressant effects of TCM are definitive and have great potential for development, but the research does contain shortcomings that may seriously constrain the development of novel antidepressants. Firstly, the hypothesis of the pathogenesis of MDD mentioned above still does not provide an adequate explanation for the nature of MDD, although current studies have elaborated the possible pathogenesis of MDD from different perspectives. At the same time, the insufficiency of basic mechanism research also restricts the deeper study of TCM antidepressants, and it is not known through which different unknown pathways TCM can still act on MDD. Secondly, TCM antidepressant studies have mainly focused on the traditional mechanisms of monoamine transmitters, HPA axis, and neurotrophic factors, and the effects of Glu and brain-gut axis on the occurrence of MDD have been well demonstrated by the studies. However, the studies of TCM in treating MDD through these pathways are still lacking in reports. In addition, compared with other drugs that have been widely used, most of the studies on TCM still remain in the preclinical research stage, and there is a lack of clinical trials to elucidate the exact antidepressant effects of TCM.

Therefore, to address the above problems, further development of basic mechanism studies is urgently needed, with a view to developing a unified understanding of the etiology and pathogenesis of MDD, exploring the antidepressant effects of TCM in depth and comprehensively, in order to elucidate the nature of MDD and the moderating effects of TCM, and providing a scientific basis for new means of treating MDD.

## Abbreviations

5-HT, serotonin; ACTH, adrenocorticotropic hormone; AVP, arginine vasopressin; BDNF, brain-derived neurotrophic factor; CMS, chronic mild stress; CNS, central nervous system; CORT, cortisol; CRH, corticotropin-releasing hormone; CRS, chronic restraint stress; CSDS, chronic socially frustrating stress; CUMS, chronic unpredictable stress; DA, dopamine; FST, forced swimming; GABA, gamma-aminobutyric acid; GDNF, glial cell line-derived neurotrophic factor; GLT-1, Glial glutamate transporter protein-1; GR, glucocorticoid recepto; HPA, Hypothalamus-Pituitary-Adrenal; IL-1, interleukin-1; IL-6, interleukin-6; MCAO, middle cerebral artery occlusion; MDD, Major Depressive Disorder/Depression; MS, maternal separation; NE, norepinephrine; NGF, nerve growth factor; NMDA, N-methyl-D-aspartic acid OB, olfactory bulbectomy; PSD, post-stroke depression; PVN, paraventricular nucleus; SCFAs, short-chain fatty acids; TCM, Traditional Chinese Medicine; TNF-α, Tumor necrosis factor-alpha; TrkB, tyrosine kinase B; TST, trailing suspended tail; VEGF, vascular endothelial growth factor.
